# PTSD, Risk of Suicide, and Unintended Death by Overdose in Late Life

**DOI:** 10.1093/geroni/igaa057.2688

**Published:** 2020-12-16

**Authors:** Amy Byers, Yixia Li, Shira Maguen, Thomas Neylan

**Affiliations:** 1 University of California, San Francisco / San Francisco VA Health Care System, San Francisco, California, United States; 2 Northern California Institute for Research and Education, San Francisco, California, United States

## Abstract

Little is known about late-life posttraumatic stress disorder (PTSD) and risk of suicide and apparent accidental death by overdose. We studied 488,044 older veterans (50 and older) with PTSD and propensity-matched comparison group without PTSD (n=488,044), seen in VA 2012-2013 followed to 12/31/2016. There were 5,693 non-fatal and fatal suicide attempts for those with PTSD and 4,310 for those without PTSD (approximately 20% fatal for each group). Those with PTSD had nearly 2-fold increased risk of any attempt [HR=1.55 (95% CI=1.49-1.62)]. While results specific to death by suicide were non-significant, impact of PTSD on method of death (e.g., drug overdose, firearms, hanging) was significant only for drug overdose [HR=1.58 (95% CI=1.22-2.03)]. Intentional and unintentional death by narcotics and such drugs as non-opioid analgesics and autonomic nervous system drugs were most highly associated with late-life PTSD. This study provides important implications for late-life suicide prevention related to PTSD and cause-specific drugs. Part of a symposium sponsored by the Aging, Alcohol and Addictions Interest Group.

